# Activation of Regulatory T Cells during Inflammatory Response Is Not an Exclusive Property of Stem Cells

**DOI:** 10.1371/journal.pone.0035512

**Published:** 2012-04-23

**Authors:** Jan-Hendrik Gosemann, Joachim F. Kuebler, Michela Pozzobon, Claudia Neunaber, Julia H. K. Hensel, Marco Ghionzoli, Paolo de Coppi, Benno M. Ure, Gesine Holze

**Affiliations:** 1 Center of Pediatric Surgery Hannover, Hannover Medical School and Bult Children's Hospital, Hannover, Germany; 2 Stem Cell Processing Laboratory, Clinic of Pediatric Oncohematology, University of Padova, Padova, Italy; 3 Trauma Department, Hannover Medical School, Hannover, Germany; 4 Surgery Unit, UCL Institute of Child Health, Great Ormond Street Hospital, London, United Kingdom; University of Medicine and Dentistry of New Jersey, United States of America

## Abstract

**Background:**

Sepsis and systemic-inflammatory-response-syndrome (SIRS) remain major causes for fatalities on intensive care units despite up-to-date therapy. It is well accepted that stem cells have immunomodulatory properties during inflammation and sepsis, including the activation of regulatory T cells and the attenuation of distant organ damage. Evidence from recent work suggests that these properties may not be exclusively attributed to stem cells. This study was designed to evaluate the immunomodulatory potency of cellular treatment during acute inflammation in a model of sublethal endotoxemia and to investigate the hypothesis that immunomodulations by cellular treatment during inflammatory response is not stem cell specific.

**Methodology/Principal Findings:**

Endotoxemia was induced via intra-peritoneal injection of lipopolysaccharide (LPS) in wild type mice (C3H/HeN). Mice were treated with either vital or homogenized amniotic fluid stem cells (AFS) and sacrificed for specimen collection 24 h after LPS injection. Endpoints were plasma cytokine levels (BD™ Cytometric Bead Arrays), T cell subpopulations (flow-cytometry) and pulmonary neutrophil influx (immunohistochemistry). To define stem cell specific effects, treatment with either vital or homogenized human-embryonic-kidney-cells (HEK) was investigated in a second subset of experiments. Mice treated with homogenized AFS cells showed significantly increased percentages of regulatory T cells and Interleukin-2 as well as decreased amounts of pulmonary neutrophils compared to saline-treated controls. These results could be reproduced in mice treated with vital HEK cells. No further differences were observed between plasma cytokine levels of endotoxemic mice.

**Conclusions/Significance:**

The results revealed that both AFS and HEK cells modulate cellular immune response and distant organ damage during sublethal endotoxemia. The observed effects support the hypothesis, that immunomodulations are not exclusive attributes of stem cells.

## Introduction

Endotoxemia, the presence of bacteria in the blood, can lead to sepsis and subsequent multiple organ dysfunction syndrome due to uncontrolled activation of the innate immune system and a resulting fulminant inflammatory cascade. Despite modern intensive care medicine with multimodal therapy including immunomodulatory agents, sepsis remains a major cause of death on intensive care units [1].

Stem cells of various origins have been widely investigated for their therapeutic potential during the last decades. Promising results from *in vitro* and *in vivo* studies resulted in a large number of clinical trials and experimental clinical applications (i.e. autoimmune diseases, neurodegenerative diseases, spinal trauma, cerebrovascular disease, cardiovascular diseases, degenerative liver disease, cancer, tissue engineering) [2], [3]. More recently, the experimental therapeutic application of stem cells during inflammation and sepsis introduced new potential strategies for critical care medicine. It is now widely accepted that stem cells provide immunomodulatory properties during inflammation and sepsis. The majority of therapeutic stem cell studies revealed anti-inflammatory immunomodulations resulting in decreased organ damage and increased survival [4], [5], [6]. The observed immunomodulations include suppression of T-lymphocytes (CD4^+^ and CD8^+^) but also induction of regulatory T cells (Tregs) [7], [8], which in turn have been reported to decrease distant organ damage [9]. However, the exact molecular backgrounds of these phenomena remain unclear and several species dependent differences have been recognized: Whereas enhancing the immunosuppressive effect of human mesenchymal stem cells (MSCs) has been reported to be mainly dependent on soluble factors, rodent MSCs require cell-to-cell contact of viable cells [7], [8], [10].

MSCs are rare *in vivo* and difficult to expand *in vitro* where they rapidly senescence [11]. Moreover, the application of MSCs may bare some risks (including malignant tumor formation) and the discussion about potential therapeutic values is controversial [12], [13].

The isolation of pluripotent stem cells from amniotic fluid (AFS cells) by selection for expression of the membrane stem cell factor receptor c-Kit, revealed a new and safe source of cells that could have a therapeutic value in various diseases prenatally and/or postnatally [14]. AFS cells are easy to isolate and in contrast to MSCs, to expand and to be genetically manipulated [15], [16]

Subsequently, the potential of AFS cell treatment has been investigated in a rat model of necrotizing enterocolitis (NEC), a severe septic condition of ischemic and inflammatory nature in mostly premature infants. Zani *et al.* reported increased survival, decreased incidence of NEC together with decreased bowel damage after intraperitoneal injection of AFS cells [17]. It is however unclear whether in that model of disease AFS cells do mediate the immunological response.

The exclusive role of stem cell triggered immunomodulation has been recently questioned, showing that mature stromal cells of various origins provide a powerful immunomodulation comparable if not superior to stem cells [18]. Moreover, very recently it has been shown that AFS cells may be able to suppress inflammatory responses *in vitro* via a paracrine/soluble factors pathway [19]. However, the role of AFS cell treatment during *in vivo* inflammatory response remains unclear.

The present study was designed to investigate the modulation of inflammatory response to lipopolysaccharide (LPS) endotoxemia by cellular treatment with AFS cells. To evaluate if possible immunomodulations are dependent on cell viability either vital or homogenized AFS cells were administered. In a second subset of experiments, human-embryonic-kidney-cells (HEK [293 T]), a non-stem cell line of epithelial origin, were administered in the same model of sublethal endotoxemia to define stem cell specific effects.

This study was designed to evaluate the hypothesis that immunomodulations by cellular treatment during inflammatory response is not stem cell specific.

## Materials and Methods

### Cell lines

#### AFS cells

Human AFS cells were received as a generous gift from Dr de Coppi. They were obtained, characterized and cultured as previously described [15]. Briefly, cells were collected from consenting volunteer donors (following guidelines of the Azienda Ospedaliera Padova [protocol number 451P/32887]) and selected by CD117 (c-Kit) immunoselection, using Mini-MACS (Miltenyi Biotec S.r.l., Bologna, Itlay) as previously described [15], [20]. Cells were subcultured routinely at a dilution of 1∶4 to 1∶8 and expansion <70% confluence was ensured.

Phenotypic fluorescence activated cell sorter (FACS) analysis of AFS cells was studied by incubating cells with anti-human antibodies: CD29 FITC, CD44 FITC, CD73 PE, CD105 PE, (Beckton Dickinson, Pharmingen, San Jose, CA); HLA-ABC FITC and HLA-DR PE (Immunotech, Marseille, France). Immunofluorescence of AFS cells in cover slip was performed. Cells were fixed in paraformaldehyde 4% for 5 minutes. Staining was performed using c-Kit antibody (Santa Cruz Sc-13508) and SSEA-4 (Santa Cruz, Biotechnology, CA, USA). Sections were mounted with 4,6-diamidino-2-phenylindole (Vector Laboratories, Segrate, Italy) and images were captured with Olympus BX60 microscope (Olympus Italia S.r.l., Segrate, Italy) using Viewfinder Lite software.

#### HEK 293 T cells

HEK 293 T is a well characterized, commercially available cell line of epithelial origin. HEK 293 T cells were purchased at ATCC, LGC Standards GmbH, Germany and cultured in Dulbecco's Modified Eagle Medium (DMEM), 1% Penicillin/Streptomycin and 5% Fetal Calf Serum. Cells were subcultured routinely at a dilution of 1∶4 to 1∶8 and expansion <70% confluence was ensured.

### Ethics statement

All animal studies were performed in accordance with the guidelines of the Federation of European Laboratory Animal Science Associations (FELASA) and the Society of Laboratory Animal Science (SLAS) and were approved by the local Institute for Laboratory Animal Research (Hannover Medical School) and the local Ministry of Consumer Protection and Food Safety of Lower Saxony, Germany (Permit Nr: 339-42502-12-10-0046).

### Animals

For all experiments in this study male C3H/HeN wild type mice (Charles River, Germany), weighing 25–30 g, were used. Mice have been handled in our animal facility with a room temperature of 20–24°C, a relative humidity of 55±5% and a light dark cycle of 12 h. Water and pelleted mouse chow (Altromin 1324) was available throughout the study period.

### Mouse model of endotoxemia and group distribution

**Figure 1:**
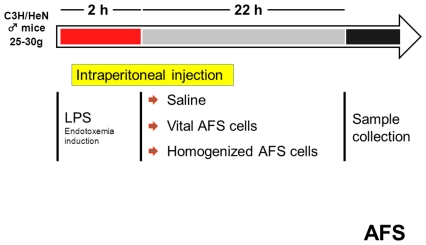
Mouse model of sublethal endotoxemia (study design). Male C3H/HeN mice were randomly assigned to either application of vital AFS cells (vitAFS), homogenized AFS cells (homAFS) or saline only (positive-control). Sublethal endotoxemia was induced by intraperitoneal injection of LPS. Mice distributed to the vitAFS group received 10^6^ vital AFS cells in 0.7 ml sterile phosphate buffered saline (PBS) intraperitoneally 2 h after LPS challenge. The same amount of cells and PBS was used for treatment of the homAFS group, but prior to injection vital cells were disrupted using the Sonopulse cell disperser (BANDELIN electronic, Berlin, Germany). The positive-control group received 0.7 ml sterile PBS without cells or disrupted cell material. Mice were sacrificed 24 h after LPS challenge and blood as well as tissue specimens were harvested. A group of 6 animals was sacrificed without any treatment (negative-control) to determine basal cytokine levels, T-cell subtype populations and lung polymorphonuclear neutrophil (PMN) infiltration.

The study design is presented in Figure 1. To induce sublethal endotoxemia, mice were injected intraperitoneally with LPS (LPS, *Escherichia coli*, Serotype 0127:B8, Sigma, 6 mg/kg) dissolved in phosphate buffered saline (PBS).

**Figure 2:**
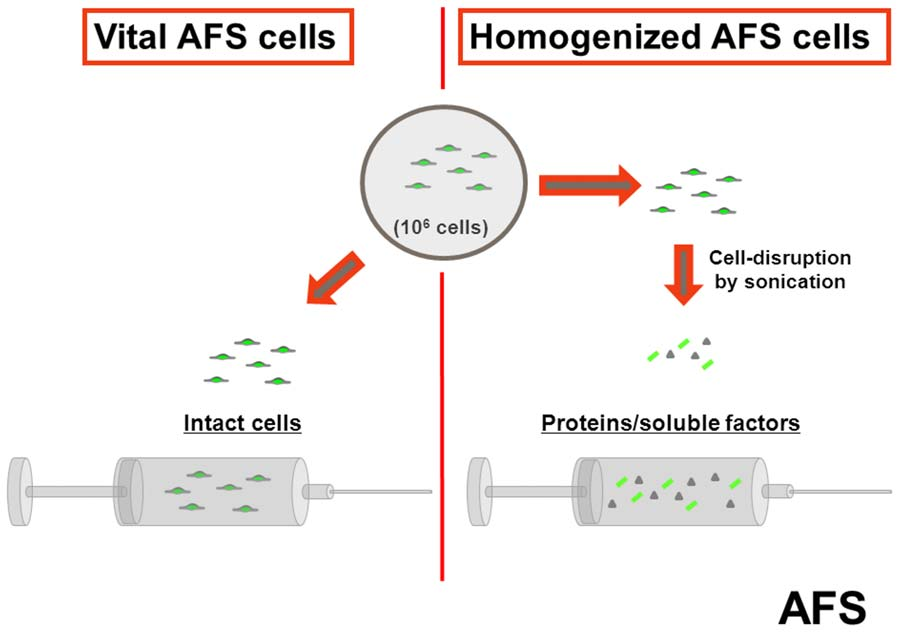
Preparation of vital AFS cells and homogenized AFS cells. 10^6^ vital AFS cells (vitAFS) were prepared in 0.7 ml sterile phosphate buffered saline (PBS) for intraperitoneal injection in mice randomly assigned to the vitAFS group (2 h after LPS challenge). For the preparation of homogenized AFS cells, 10^6^ vital AFS cells underwent 5 min cell-disruption in 0.7 ml PBS prior to injection by sonication.

Mice were randomly assigned to either application of vital AFS cells (vitAFS), homogenized AFS cells (homAFS) or saline only (positive-control). Mice distributed to the vitAFS group received 10^6^ vital AFS cells in 0.7 ml sterile phosphate buffered saline (PBS) intraperitoneally 2 h after LPS challenge. The same amount of cells and PBS was used for treatment of the homAFS group, but prior to injection vital cells were disrupted by sonication (5 min), using the Sonopulse cell disperser (BANDELIN electronic, Berlin, Germany). The positive-control group received 0.7 ml sterile PBS without cells or disrupted cell material (Figure 2).

Eight hours after LPS injection 6 animals out of each group were sacrificed to determine acute cytokine response. The remaining animals (n = 18 per group) were sacrificed 24 h after LPS challenge and blood as well as tissue specimens were harvested.

To determine stem cell specific effects, a second set of experiments was performed with vital (vitHEK) as well as homogenized (homHEK) human embryonic kidney cells (HEK 293 T, ATCC, LGC Standards GmbH, Germany) according to the same protocol (n = 6 per group), which was used for AFS cells. A group of 6 animals was sacrificed without any treatment (negative-control) to determine basal cytokine levels, T-cell subtype populations and lung polymorphonuclear neutrophil (PMN) infiltration. All animals were clinically observed and moribund mice were euthanatized immediately.

### Sample collection (Plasma, Lymphocytes, Lung tissue)

Mice were anesthetized with 100 mg/kg Ketamine (Ketanest®, Pfizer Pharma, Germany) and 5 mg/kg Xylazine (Rompun®, Bayer, Germany). They were exsanguinated by cardiac puncture. Heparinized blood was centrifuged (3000 U/min, 5 min, 4°C), plasma was snap-frozen in liquid nitrogen and stored at −80°C. Lymphocytes were extracted by straining the spleen through a mesh. The obtained cells were resuspended in 0.9% sterile saline. Cells were washed twice with phosphate-buffered saline (PBS), diluted in 1 mL 0.9% sterile saline and processed for the evaluation of CD4^+^, CD8^+^ and CD4+CD25+Foxp3^+^ cells as described below.

Lung tissue was harvested and fixed in 4% formalin for 24 h followed by paraffin embedding for histological evaluation.

### Assessment of T-cell subtypes (CD4^+^, CD8^+^) and CD4+CD25+Foxp3^+^ Tregs

Remaining erythrocytes in the cell suspension were treated with erythrocyte lysis buffer (0.16 M NH_4_Cl, 11.9 M KHCO_3_ and 0.27 M ethylene diamine tetraacetic acid [EDTA]) for 15 min. The leukocyte fraction was resuspended in PBS after washing twice. Antibodies to detect CD4^+^ (PE, BD Pharmingen, San Diego, CA), CD8^+^ (FITC, BD Pharmingen, San Diego, CA), CD25^+^ (PE-Cy7, BD Pharmingen, San Diego, CA) and FoxP3^+^ (APC, eBioscience, Frankfurt, Germany) cells were used. Cells were stained as previously described [21]. Intracellular staining of *forkhead box protein 3* (Foxp3), a well-established Treg marker, was performed as described elsewhere [22]. A FACSCanto cytometer (Becton Dickinson, Heidelberg, Germany) was used for detection. Data analysis was carried out using FlowJo Software (Tree Star Inc., Ashland, OR).

### Assessment of plasma cytokine content

#### Cytometric Bead Array

*Monocyte chemoattractant protein-1* (MCP-1), *interleukin-6* (IL-6), *tumor necrosis factor*-α (TNF-α) and *interleukin-10* (IL-10) concentrations in plasma were determined with cytometric bead arrays using the BD™ CBA Mouse Inflammation Kit (BD Pharmingen, San Diego, CA, USA) according to the manufacturer's instruction. In brief, 50 µl of mixed capture beads and 50 µL sample were incubated for 1 h and after washing 50 µl *Mouse Inflammation PE Detection Reagent* was added. After incubation for 1 h, complexes were washed and analyzed using a FACSCalibur Cytometer (BD Biosciences, Heidelberg, Germany). Data analysis was carried out using BD CellQuest™ Software.

#### IL-2 and TGF-β ELISA

*Interleukin-2* (IL-2) and *transforming growth factor-beta* (TGF-β) plasma levels were determined by enzyme-linked immunosorbent assay (ELISA), using the Platinum ELISA system precoated with primary antibodies against IL-2 and TGF-β according to the manufacturer's instructions (eBioscience, Frankfurt, Germany). Briefly, Plasma was diluted either 1∶2 (IL-2) or 1∶500 (TGF-β), applied to precoated 96well-plates in duplicates and incubated with Biotin-Conjugate for either 1 h (TGF-β) or 2 h (IL-2) at room temperature. Wells were washed (5×) and horseradish-peroxidase-linked streptavidin was applied to all wells. After incubation for 1 h, wells were washed (5×) and the chromogen 3,3′,5,5′-Tetramethylbenzidine was applied for either 10 (IL-2) or 30 min (TGF-β). The chromogenic reaction was stopped by adding sulfuric acid. Wells were read with a multiplate reader (BioTek Synergy MX, Biotek, Bad Friedrichshall, Germany) at 450 nm. A standard dilution using standards with known protein concentrations was included in the ELISA procedure to calculate IL-2 and TGF-β plasma concentrations from an individual standard curve.

### Assessment of lung PMN infiltration

The lung PMN infiltration was determined by immunohistochemical staining of paraffin embedded tissue sections with anti-mouse Ly-6G primary antibody (BD Pharmingen, Heidelberg, Germany). After microwave treatment in citrate buffer (10 mM citric acid, pH 6.0) and inhibition of endogenous peroxidases with 3% hydrogen peroxidase (Merck, Darmstadt, Germany) sections were incubated with the primary Ly-6G antibody for one hour. Normal rabbit serum was used to avoid non-specific antibody binding. After washing, a horseradish peroxidase (HRP)-conjugated antibody (Dako, Hamburg, Germany) was used as secondary antibody. Immunostaining was developed by diaminobenzidine (Dako, Hamburg, Germany) and slides were counterstained with Mayer's haemalaum. For evaluation of the number of infiltrating neutrophils, sections were blinded and randomized. Ly-6G^+^ neutrophils were counted in four representative, non-overlapping high-power fields per section (Leica DM LB, Leica Microsystems, Wetzlar, Germany).

### Statistics

All statistical analyses were performed using Sigma-Stat computer software (SPSS Inc., Chicago, IL, USA). We compared groups using one-way analysis of variance (ANOVA) followed by either Scheffe's or Tukey's post-hoc test for multiple comparison. Differences were considered significant at a p-value of less than 0.05 and results are expressed as mean ± standard error of the mean (SEM).

## Results

### AFS cell culture

#### AFS cell characterization

**Figure 3:**
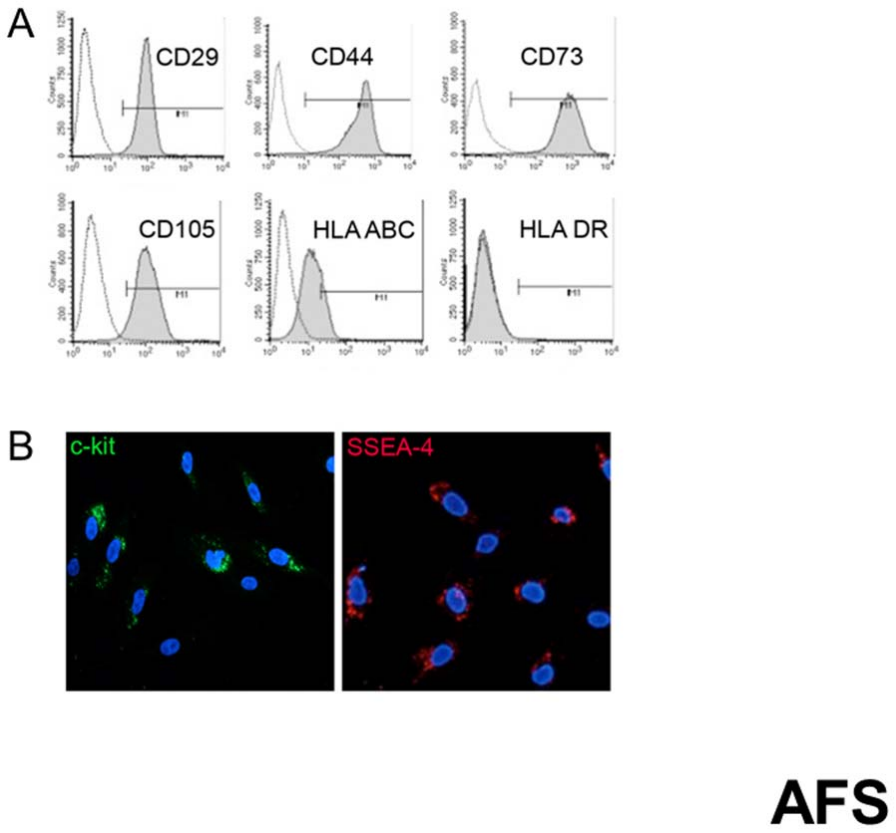
Characterization of AFS cells by FACS analysis and Immunofluorescence. A. Representative histograms by fluorescence activated cell sorter (FACS) analysis showing the expression of CD29, CD44, CD73,CD105, HLA-ABC and DR of AFS cells. The respective isotype control is shown as a gray line. B. Representative immunofluorescence of AFS cells showing positive staining for c-kit and SSEA-4. Real magnification: 20×.

Phenotypic FACS analysis underlined that human AFS cells stably expressed CD29, CD44 (hyaluronate receptor), and stromal cell markers such as CD90 (Thy-1), CD105 (endoglin, TGF-β receptor), and CD73 at different passages (analysis performed until passage 8). Human AFS cells also expressed HLA-ABC but not HLA-DR, indicating low immunogenicity profile. The expression of c-Kit was analyzed at different passages by immunofluorescence showing that the surface marker is internalized (present until passage 8), importantlty SSEA-4 (stem cell embryonic antigen-4) was consistently expressed (Figure 3).

### AFS application

#### Plasma cytokine response 8 h after LPS challenge

Basal IL-6 plasma concentrations of non-treated negative-controls were not detectable and considered 0 pg/ml. 8 h after intraperitoneal LPS injection (6 h after treatment with either vitAFS, homAFS, or saline-treated positive-controls) IL-6 plasma levels were significantly increased in all endotoxemic groups (range: 1546.89 to 5855.86 pg/ml) compared to negative-controls. Multi comparison revealed no significant difference between positive-controls and vitAFS or homAFS.

#### Plasma cytokine response 24 h after LPS challenge

**Figure 4:**
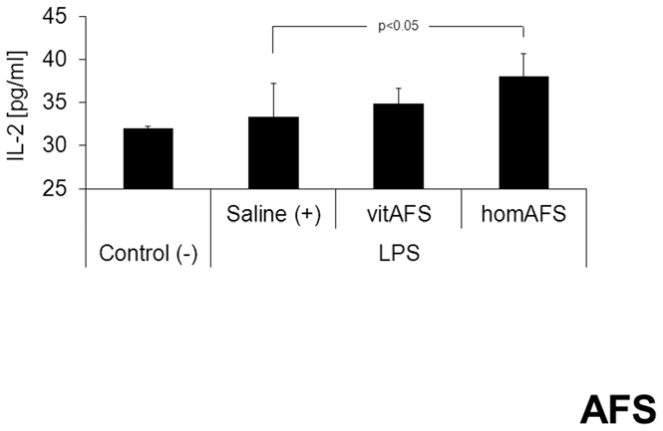
IL-2 plasma levels after AFS application. The IL-2 plasma levels were significantly increased in the group treated with homAFS compared to saline-treated positive-controls (+).

Negative-controls showed basal IL-6, TNF-α, MCP-1 and IL-10 plasma concentrations of 0 pg/ml. Plasma cytokine levels of the above mentioned cytokines/chemokines were still altered 24 h after LPS injection compared to negative-controls, but no significant difference was detected throughout the two AFS-treated groups compared to the positive control as shown in table 1. TGF-β plasma levels were not altered throughout the groups (data not shown). The IL-2 plasma levels were significantly increased in the group treated with homAFS compared to saline-treated positive-controls (Figure 4).

**Table 1:** Plasma cytokine response 24 h after LPS challenge (AFS cells).

	Control (−)	LPS		
		Saline (+)	Vital AFS	Hom. AFS
IL-6 [pg/ml]	0	9.99±1.44*	12.63±2.05*	12.28±2.79*
TNF-α [pg/ml]	0	18.88±1.05*	17.08±0.93*	17.65±1.3*
MCP-1[pg/ml]	0	448.94±47.74*	498.1±87.47*	416.39±46.05*
IL-10 [pg/ml]	0	8.09±1.26*	8.48±0.83*	8.99±1.65*

* p<0.05 vs. Control.

#### Lymphocyte subpopulations

**Figure 5:**
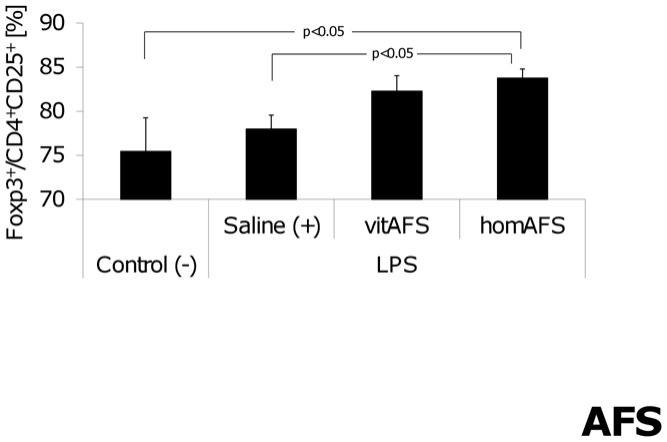
Percentage of regulatory T cells after AFS cell application. The percentage of Foxp3^+^ lymphocytes was significantly increased in the pool of CD4^+^CD25^+^ lymphocytes after application of homAFS compared to saline-treated positive-controls (+) and non-endotoxemic negative-controls (−).

The percentage of CD4^+^CD25^+^ lymphocytes expressing Foxp3^+^ (Tregs) was significantly increased in the group treated with homAFS compared to saline-treated positive-controls and non-endotoxemic negative-controls. Mice treated with vitAFS tended to show similar regulations but the differences were not significant (Figure 5). No changes in CD4^+^ and CD8^+^ lymphocyte distribution were observed throughout the endotoxemic groups compared to negative-controls 24 h after LPS challenge (data not shown).

#### Pulmonary PMN infiltration

**Figure 6:**
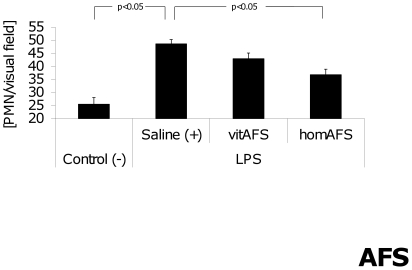
Pulmonary PMN infiltration after AFS cell application. Pulmonary infiltration with PMNs was significantly increased in positive-controls (+) compared to negative-controls (−) 24 h after LPS injection. Mice treated with hom AFS cells showed significantly decreased pulmonary neutrophil influx compared to positive-controls (+).

Pulmonary infiltration with neutrophils was significantly increased in positive-controls compared to negative-controls 24 h after LPS injection. In contrast, homAFS application resulted in significantly decreased pulmonary neutrophil infiltration compared to positive-controls. No significant difference was found between homAFS-treated mice compared to negative-controls. Again, vitAFS-treated animals showed similar effects but statistical significance was not reached (Figure 6).

### HEK 293 T application

#### Plasma cytokine levels 24 h after LPS challenge

**Figure 7:**
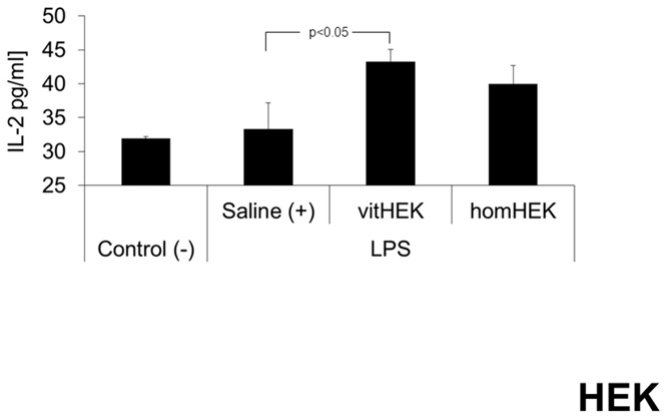
IL-2 plasma levels after HEK application. The IL-2 plasma levels were significantly increased in the group treated with vitHEK compared to saline-treated positive-controls (+).

Parallel to the findings in the AFS treatment group, elevations in plasma cytokine levels of IL-6 (range 3.7 to 5.7 pg/ml), TNF-α (range 18.87 to 21.72 pg/ml) and MCP-1 (range 452.8 to 467.97 pg/ml) were still present 24 h after LPS injection compared to negative-controls. No relevant differences between the three endotoxemic groups were detected between IL-6, TNF-α, MCP-1 and TGF-β concentrations. The IL-2 plasma levels were significantly increased in the group treated with vitHEK compared to saline-treated positive-controls (Figure 7).

#### Lymphocyte subpopulations

**Figure 8:**
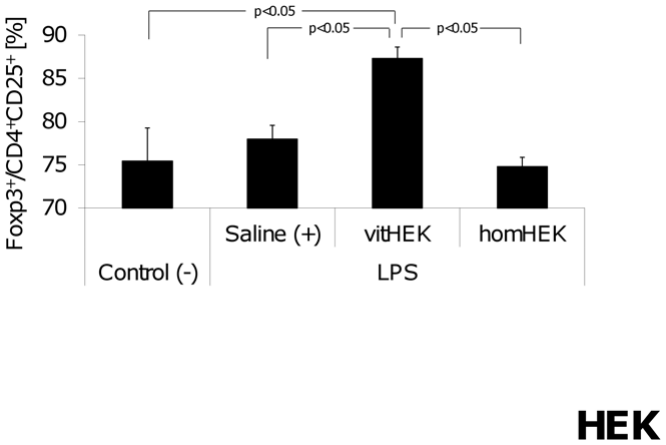
Percentage of regulatory T cells after HEK cell application. Relative expression of Foxp3 in CD4^+^CD25^+^ lymphocytes was significantly increased in the group treated with vitHEK cells compared to endotoxemic mice, which received homHEK cells, saline-treated positive-controls (+) and non-endotoxemic negative-controls (−).

Parallel to the findings after application of homAFS cells, endotoxemic mice receiving vitHEK showed a significant increase in the CD4^+^CD25^+^Foxp3^+^ regulatory T-cell population compared to negative-controls, endotoxemic saline-treated positive-controls as well as homHEK treated mice. In contrast, no alterations were found in homHEK-treated mice compared to controls (Figure 8). Distribution of CD4^+^ and CD8^+^ lymphocytes was not altered in the endotoxemic groups compared to negative-controls 24 h after LPS challenge (data not shown).

#### Pulmonary PMN infiltration

**Figure 9:**
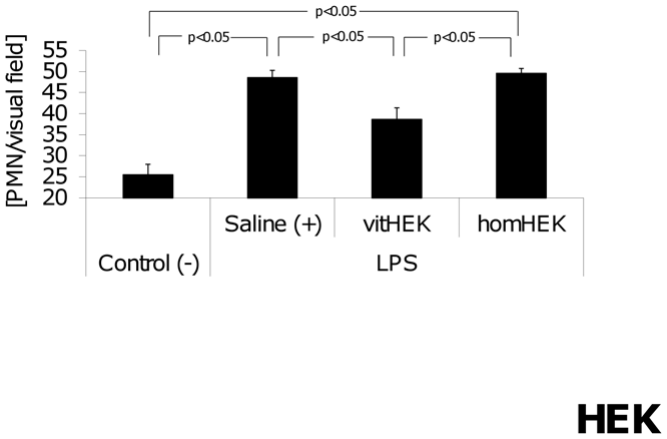
Pulmonary PMN infiltration after HEK cell application. Pulmonary infiltration with PMNs was significantly increased in positive controls (+) and homHEK (p<0.05 vs. [−]) treated endotoxemic mice compared to negative-controls (−) 24 h after LPS injection. Mice treated with vitHEK cells showed significantly decreased pulmonary neutrophil influx compared to positive-controls (+) and homHEK treated endotoxemic mice.

24 h after LPS injection, pulmonary neutrophil infiltration was significantly increased in positive-controls and homHEK-treated mice compared to negative-controls. In contrast, mice injected with vitHEK revealed decreased pulmonary neutrophil counts compared to the two other endotoxemic groups (Figure 9).

## Discussion

### Modulation of inflammatory response by AFS cells

Tregs, a unique subset of CD4^+^ lymphocytes, display potent immunoregulatory functions *in vitro* and *in vivo* [23], [24]. The immunomodulatory potential of Tregs has been reported in various studies of antigen-driven, T-cell-dependent adaptive but also antigen-independent innate immune responses [25]. Research for therapeutic implications is on-going. During infection and inflammation Tregs play an ambivalent role by inhibiting anti-microbial T effector cells and dampening immune responses [26]: On the one hand clearance of microbial contamination requires an appropriate immune reaction with significant effector cell activity; on the other hand uncontrolled immune responses may lead to distant organ damage, sepsis and multiple organ failure. Several groups investigated the role of Tregs during inflammation and sepsis. However, the exact molecular mechanisms, how Tregs provide immunomodulatory properties, remain unclear.

It is well accepted that MSCs are potent mediators of T-cell responses - including the activation of Tregs [7], [8] - and eventually provide potent anti-inflammatory properties during inflammation and sepsis [4], [7]. However, MSCs are rare *in vivo*, difficult to be expanded *in vitro*, and malignant tumor formation could be related to extensive passages in culture [13], [27], [28], [29].

Amniotic fluid stem cells have been introduced as a relatively new source of pluripotent stem cells. In contrast to MSCs, no tumor formation has been reported using AFS cells even after multiple passages [15]. The latter may be related to the fact that, differently to AFS cells, MSCs do get exposed to viruses and toxic agents during life [30].

Several possible applications in different models of disease have been evaluated to date. However, the role of AFS cells during inflammatory response remains unclear.

In our model of endotoxemia, application of homogenized AFS cells significantly increased the population of CD4^+^CD25^+^Foxp3^+^ regulatory T cells 24 h after endotoxemic challenge, whereas vital AFS cells only tended to show these effects. These findings suggest that similar to the effects reported in human MSCs, human AFS cell application during early endotoxemia induces regulatory T cell activity. Taken together, it is tempting to speculate that Treg-inducing soluble factors are highly concentrated in the homogenized AFS cell suspension after cell disruption and therefore immediately available for Treg recruitment. In contrast, vital AFS cells may provide a controlled release of these factors. Hence, a significant effect of vital AFS cell application on Treg stimulation could not be seen 24 h after LPS injection. However, parallel to the immunosuppressive effects of human MSCs [7], the modulations observed in the present study seem to be mainly controlled by soluble factors. IL-2 and TGF-β are two cytokines well known for their role in Treg induction and maintenance [24], [26], [32]. The finding of increased IL-2 levels in those endotoxemic animals, treated with homAFS cells suggests a prominent role of IL-2 for Treg induction in this setting of sublethal endotoxemia, whereas TGF-β does not seem to play a major role in our model. Due to the complex regulations and numerous possible activation pathways for Tregs [31] we believe, that one cannot attribute the activation seen in our model to only one cytokine. The IL-2 pathway represents a major but not exclusive mechanism for the development and function of Tregs [32]. Various factors (such as *human leucocyte antigen-G5* [HLA-G5], *transforming growth factor-beta* [TGF-β], *interleukin-2* [IL-2], *prostaglandin E2* [PGE2], *hepatocyte growth factor* [HGF], *interleukin-10* [IL-10], *galectin-3*) but also cell-to-cell interaction are held responsible for Treg induction [24], [33]. To date the exact molecular basis of Treg-activation remains unclear and is the focus of on-going research [24].

Heuer *et al.* reported that adoptive transfer of Tregs has been shown to improve survival and increase bacterial clearance in an animal model of polymicrobial sepsis [34]. In a model of acute lung injury D'Alessio *et al.* concluded that modulation of innate immune responses is central to the Treg mediated coordination of this response [23]. More recently, Venet *et al.* investigated the role of Tregs during indirect acute lung injury in a model of hemorrhagic shock followed by polymicrobial sepsis. The authors described a central role of Tregs to the control of neutrophil recruitment. Furthermore, they showed that specific down regulation of Treg function (Foxp3 targeting siRNA) resulted in increased pulmonary neutrophil recruitment and subsequent organ damage [9].

In our model of sublethal endotoxemia increased pulmonary neutrophil recruitment was observed in endotoxemic positive-controls 24 h after LPS challenge. Application of homAFS cells resulted in decreased neutrophil infiltration in lungs of endotoxemic mice compared to positive-controls. The parallel finding of increased Foxp3^+^ Tregs supports the hypothesis that regulatory T cells play a major role in mediating resolution of acute lung injury [23].

Gonzales Rey *et al.* reported a significant decrease in proinflammatory serum cytokines in mice suffering from polymicrobial sepsis for 18 h (CLP) and treated with human adipose tissue derived stem cells (ASC) 4 h after sepsis induction. Similar non-major histocompatibility complex (MHC)-restricted immunomodulations were reported by other groups [4], [35], [36]. In contrast to these findings, we observed only minor changes in the investigated systemic humoral immune responses after treatment with both homogenized and vital AFS cells compared to saline treatment 8 h and 24 h after sublethal LPS challenge. We chose a model of sublethal endotoxemia to avoid uncontrolled immune reactions. The present results suggest that AFS cells have no major impact on the early cytokine release in our model of endotoxemia. However, it remains unclear if or to what extent AFS cell treatment is able to prevent/influence mortality in lethal models of endotoxemia or polymicrobial sepsis. Furthermore, no changes in CD4^+^ CD8^+^ subpopulations were observed, which is probably due to the investigated time point. We assume that possible immunomodulations of T-helper cell and cytotoxic T cell activity might have a later onset.

In summary, application of AFS cells during LPS endotoxemia modulates cellular immune responses and distant organ damage. The observed immunomodulations are not dependent on viable cells, suggesting a prominent role of soluble factors. One might speculate that (once factors are identified and characterized) cytokine therapy would be very attractive following these results, but similarly to previous studies in other systems such as the heart or the kidney it is difficult to mimic precisely the various molecules and proteins secreted by the cells in that particular context [20], [37]. The cells/homogenates may therefore be necessary as well in the clinical scenario. However, it is possible that established cell lines could be used for therapy in various diseases. This approach could be facilitated by the fact that in this context stem cell engraftment does not usually persist with time and allogenic derived cells could be used for therapy [38].

### Immunomodulations are not exclusive attributes of stem cells

Initially, the *in vitro* and *in vivo* immunosuppressive effect on virtually all cells of the immune system has been exclusively attributed to MSCs [18], [39], [40]. More recently, the exclusive role of stem cells in cellular triggered immunomodulations has been challenged by several authors, demonstrating that other cell types like mature stromal cells (SCs) or renal tubular epithelial cells show similar immunosuppressive properties and are able to modulate T-cell responses and cytokine secretion *in vitro* [18], [41]. Furthermore, hepatic stellate cells have recently been reported to induce Foxp3^+^ Tregs in a setting of co-transplantation with hepatocytes [42], [43]. According to Jones *et al.* the observed effects required licensing of SCs by cell-to-cell contact to produce soluble factors, which ultimately mediate the immunosuppressive modulations [18].

In light of these findings we performed a second subset of experiments using the non-stem cell line HEK 293 T in the above-described model of endotoxemia instead of AFS cells. Interestingly, Treg activation was also observed after treatment with vital HEK, but not homogenized HEK cells. Parallel to these findings, application of vital HEK cells resulted in significantly increased IL-2 plasma levels and decreased pulmonary neutrophil infiltration compared to positive controls. Similar to the results observed in AFS treated animals, no further relevant changes in plasma cytokine concentrations or CD4^+^ CD8^+^ lymphocyte subpopulations of HEK treated animals were found. Further studies are needed to clarify the complex molecular background of the observed phenomena and to evaluate the suppressive potential of AFS and HEK on CD4^+^/CD8^+^ populations.

Our findings show that not only homogenized AFS cells but also vital HEK cells modulate cellular immune response and distant organ damage during endotoxemia. In the present study, viable HEK cells are needed for the observed immunomodulations, which is in line with the findings of Jones *et al.* [18]. Overall, these findings support the hypothesis, that immunomodulations are not exclusive attributes of stem cells.

Together with previous studies, the present study highlights the increasing evidence that to date unidentified soluble factors seem to be major contributors to the immunomodulations, previously exclusively attributed to stem cells.

### Conclusions

Both AFS and HEK cells modulate cellular immune response and distant organ damage during sublethal endotoxemia. The observed effects cannot be exclusively attributed to AFS cells and the immunomodulatory potency of cellular treatment seems to be based on isolated factors.

However, the simultaneous findings of regulatory T-cell activation and decreased pulmonary neutrophil influx suggest a previously observed role of this T-cell subpopulation in controlling neutrophil action during early inflammation. Further studies are needed to evaluate molecular basis and therapeutic potential of Treg activation during inflammatory response with regard to the crucial balance between protection and pathology.
